# On gender and stroop effect. The REM-ACT study: Acceptance and commitment therapy versus a mindfulness-based emotional regulation intervention in anxiety disorders. A randomized controlled trial

**DOI:** 10.1192/j.eurpsy.2021.2081

**Published:** 2021-08-13

**Authors:** T. Castellanos-Villaverde, E. Fernández-Jiménez, E. Vidal-Bermejo, I. Torrea-Araiz, G. Navarro-Oliver, A. Hospital-Moreno

**Affiliations:** 1 Department Of Psychiatry, Clinical Psychology And Mental Health, La Paz University Hospital, Madrid, Spain; 2 Idipaz, Department Of Psychiatry, Clinical Psychology And Mental Health, La Paz University Hospital, Madrid, Spain

**Keywords:** Mindfulness-based Emotional Regulation, Stroop effect, acceptance and commitment therapy, randomized controlled trial

## Abstract

**Introduction:**

Results about the effects of mindfulness training on the executive function of inhibition are mixed. Research about interventions in anxiety disorders is needed to exam the differential efficacy among men and women, and the factors involved in those potential gender differences.

**Objectives:**

To compare the effectiveness of Acceptance and Commitment Therapy (ACT) versus a Mindfulness-based Emotional Regulation (MER) intervention on inhibitory control according to gender.

**Methods:**

This study was carried out in a Mental Health Unit in Spain (Colmenar Viejo, Madrid). Firstly, 80 adult patients with anxiety disorders were randomized according to the score on the Acceptance and Action Questionnaire-II (blocking factor), of whom, 64 patients decided to participate (mean age = 40.66, S.D. = 11.43; 40 females). Each intervention was weekly, during 8 weeks, guided by two Clinical Psychology residents. A 2x2x2 mixed ANOVA (pre-post change x intervention type x gender) was conducted, with Sidak-correction post-hoc tests. The dependent variable was the Interference score of the Stroop test.

**Results:**

Normality and homoscedasticity assumptions were met. No statistically significant differences were observed on age or gender between interventions. A statistically significant interaction effect was observed between pre-post change x intervention x gender on Interference [F_(1, 52)_ = 5.004, p = .030; statistical power observed = 59.3%]. Improvement in interference was larger for women after ACT (p = .000) and for men after MER (p = .002).

**Conclusions:**

These preliminary results show improvements in inhibition after the two interventions examined. However, each treatment maximizes improvement in different ways according to gender. Further research is required.

**Disclosure:**

No significant relationships.

